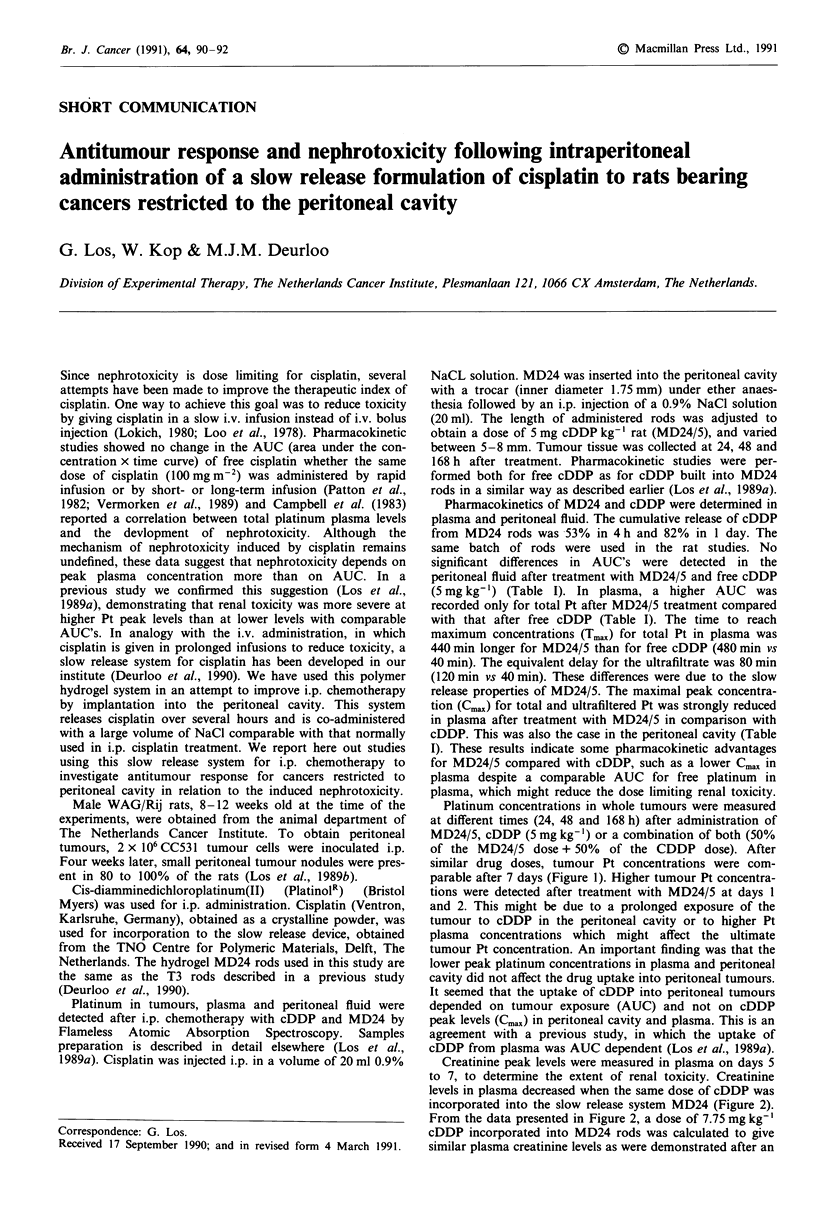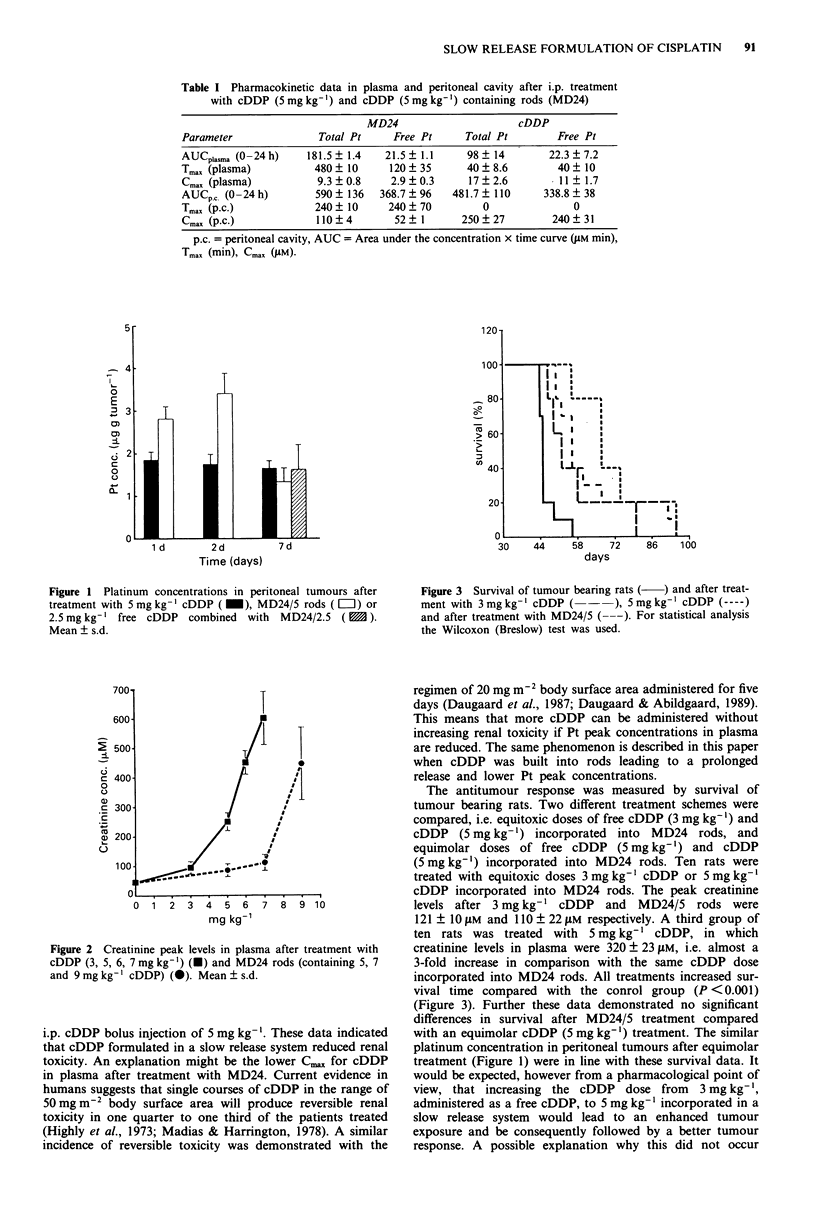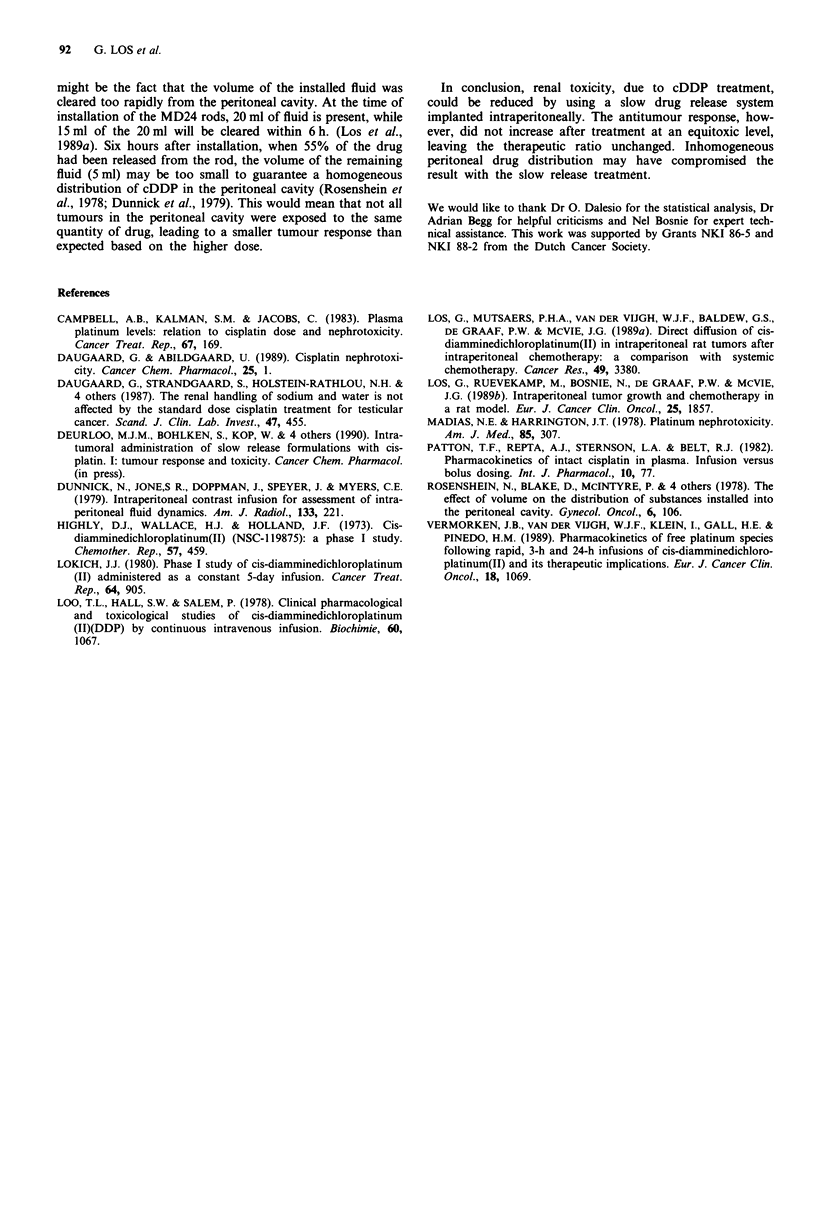# Antitumour response and nephrotoxicity following intraperitoneal administration of a slow release formulation of cisplatin to rats bearing cancers restricted to the peritoneal cavity.

**DOI:** 10.1038/bjc.1991.246

**Published:** 1991-07

**Authors:** G. Los, W. Kop, M. J. Deurloo

**Affiliations:** Division of Experimental Therapy, The Netherlands Cancer Institute, Amsterdam.


Br. J. Cancer (1991), 64, 90-92 © Macmillan Press Ltd., 1991

SHORT COMMUNICATION

### Antitumour response and nephrotoxicity following intraperitoneal administration of a slow release formulation of cisplatin to rats bearing cancers restricted to the peritoneal cavity

G. Los, W. Kop & M.J.M. Deurloo

*Division of Experimental Therapy, The Netherlands Cancer Institute, Plesmanlaan 121, 1066 CX Amsterdam, The Netherlands.*

Since nephrotoxicity is dose limiting for cisplatin, several attempts have been made to improve the therapeutic index of cisplatin. One way to achieve this goal was to reduce toxicity by giving cisplatin in a slow i.v. infusion instead of i.v. bolus injection (Lokich, 1980; Loo *et al.*, 1978). Pharmacokinetic studies showed no change in the AUC (area under the concentration × time curve) of free cisplatin whether the same dose of cisplatin ($100\,mg\,m^{-2}$) was administered by rapid infusion or by short- or long-term infusion (Patton *et al.*, 1982; Vermorken *et al.*, 1989) and Campbell *et al.* (1983) reported a correlation between total platinum plasma levels and the devlopment of nephrotoxicity. Although the mechanism of nephrotoxicity induced by cisplatin remains undefined, these data suggest that nephrotoxicity depends on peak plasma concentration more than on AUC. In a previous study we confirmed this suggestion (Los *et al.*, 1989*a*), demonstrating that renal toxicity was more severe at higher Pt peak levels than at lower levels with comparable AUC's. In analogy with the i.v. administration, in which cisplatin is given in prolonged infusions to reduce toxicity, a slow release system for cisplatin has been developed in our institute (Deurloo *et al.*, 1990). We have used this polymer hydrogel system in an attempt to improve i.p. chemotherapy by implantation into the peritoneal cavity. This system releases cisplatin over several hours and is co-administered with a large volume of NaCl comparable with that normally used in i.p. cisplatin treatment. We report here out studies using this slow release system for i.p. chemotherapy to investigate antitumour response for cancers restricted to peritoneal cavity in relation to the induced nephrotoxicity.

Male WAG/Rij rats, 8–12 weeks old at the time of the experiments, were obtained from the animal department of The Netherlands Cancer Institute. To obtain peritoneal tumours, $2 \times 10^6$ CC531 tumour cells were inoculated i.p. Four weeks later, small peritoneal tumour nodules were present in 80 to 100% of the rats (Los *et al.*, 1989*b*).

Cis-diamminedichloroplatinum(II) (Platinol$^R$) (Bristol Myers) was used for i.p. administration. Cisplatin (Ventron, Karlsruhe, Germany), obtained as a crystalline powder, was used for incorporation to the slow release device, obtained from the TNO Centre for Polymeric Materials, Delft, The Netherlands. The hydrogel MD24 rods used in this study are the same as the T3 rods described in a previous study (Deurloo *et al.*, 1990).

Platinum in tumours, plasma and peritoneal fluid were detected after i.p. chemotherapy with cDDP and MD24 by Flameless Atomic Absorption Spectroscopy. Samples preparation is described in detail elsewhere (Los *et al.*, 1989*a*). Cisplatin was injected i.p. in a volume of 20 ml 0.9% NaCL solution. MD24 was inserted into the peritoneal cavity with a trocar (inner diameter 1.75 mm) under ether anaesthesia followed by an i.p. injection of a 0.9% NaCl solution (20 ml). The length of administered rods was adjusted to obtain a dose of $5\,mg$ cDDP $kg^{-1}$ rat (MD24/5), and varied between 5–8 mm. Tumour tissue was collected at 24, 48 and 168 h after treatment. Pharmacokinetic studies were performed both for free cDDP as for cDDP built into MD24 rods in a similar way as described earlier (Los *et al.*, 1989*a*).

Pharmacokinetics of MD24 and cDDP were determined in plasma and peritoneal fluid. The cumulative release of cDDP from MD24 rods was 53% in 4 h and 82% in 1 day. The same batch of rods were used in the rat studies. No significant differences in AUC's were detected in the peritoneal fluid after treatment with MD24/5 and free cDDP ($5\,mg\,kg^{-1}$) (Table I). In plasma, a higher AUC was recorded only for total Pt after MD24/5 treatment compared with that after free cDDP (Table I). The time to reach maximum concentrations ($T_{max}$) for total Pt in plasma was 440 min longer for MD24/5 than for free cDDP (480 min *vs* 40 min). The equivalent delay for the ultrafiltrate was 80 min (120 min *vs* 40 min). These differences were due to the slow release properties of MD24/5. The maximal peak concentration ($C_{max}$) for total and ultrafiltered Pt was strongly reduced in plasma after treatment with MD24/5 in comparison with cDDP. This was also the case in the peritoneal cavity (Table I). These results indicate some pharmacokinetic advantages for MD24/5 compared with cDDP, such as a lower $C_{max}$ in plasma despite a comparable AUC for free platinum in plasma, which might reduce the dose limiting renal toxicity.

Platinum concentrations in whole tumours were measured at different times (24, 48 and 168 h) after administration of MD24/5, cDDP ($5\,mg\,kg^{-1}$) or a combination of both (50% of the MD24/5 dose + 50% of the CDDP dose). After similar drug doses, tumour Pt concentrations were comparable after 7 days (Figure 1). Higher tumour Pt concentrations were detected after treatment with MD24/5 at days 1 and 2. This might be due to a prolonged exposure of the tumour to cDDP in the peritoneal cavity or to higher Pt plasma concentrations which might affect the ultimate tumour Pt concentration. An important finding was that the lower peak platinum concentrations in plasma and peritoneal cavity did not affect the drug uptake into peritoneal tumours. It seemed that the uptake of cDDP into peritoneal tumours depended on tumour exposure (AUC) and not on cDDP peak levels ($C_{max}$) in peritoneal cavity and plasma. This is an agreement with a previous study, in which the uptake of cDDP from plasma was AUC dependent (Los *et al.*, 1989*a*).

Creatinine peak levels were measured in plasma on days 5 to 7, to determine the extent of renal toxicity. Creatinine levels in plasma decreased when the same dose of cDDP was incorporated into the slow release system MD24 (Figure 2). From the data presented in Figure 2, a dose of $7.75\,mg\,kg^{-1}$ cDDP incorporated into MD24 rods was calculated to give similar plasma creatinine levels as were demonstrated after an

Correspondence: G. Los.
Received 17 September 1990; and in revised form 4 March 1991.

**Table I** Pharmacokinetic data in plasma and peritoneal cavity after i.p. treatment with cDDP (5 mg $kg^{-1}$) and cDDP (5 mg $kg^{-1}$) containing rods (MD24)

| | *MD24* | | *cDDP* | |
|---|---|---|---|---|
| *Parameter* | *Total Pt* | *Free Pt* | *Total Pt* | *Free Pt* |
| $AUC_{plasma}$ (0–24 h) | 181.5 ± 1.4 | 21.5 ± 1.1 | 98 ± 14 | 22.3 ± 7.2 |
| $T_{max}$ (plasma) | 480 ± 10 | 120 ± 35 | 40 ± 8.6 | 40 ± 10 |
| $C_{max}$ (plasma) | 9.3 ± 0.8 | 2.9 ± 0.3 | 17 ± 2.6 | 11 ± 1.7 |
| $AUC_{p.c.}$ (0–24 h) | 590 ± 136 | 368.7 ± 96 | 481.7 ± 110 | 338.8 ± 38 |
| $T_{max}$ (p.c.) | 240 ± 10 | 240 ± 70 | 0 | 0 |
| $C_{max}$ (p.c.) | 110 ± 4 | 52 ± 1 | 250 ± 27 | 240 ± 31 |

p.c. = peritoneal cavity, AUC = Area under the concentration × time curve (µM min), $T_{max}$ (min), $C_{max}$ (µM).

**Figure 1** Platinum concentrations in peritoneal tumours after treatment with 5 mg $kg^{-1}$ cDDP (■), MD24/5 rods (□) or 2.5 mg $kg^{-1}$ free cDDP combined with MD24/2.5 (▨). Mean ± s.d.

**Figure 3** Survival of tumour bearing rats (——) and after treatment with 3 mg $kg^{-1}$ cDDP (— — —), 5 mg $kg^{-1}$ cDDP (----) and after treatment with MD24/5 (—-—). For statistical analysis the Wilcoxon (Breslow) test was used.

**Figure 2** Creatinine peak levels in plasma after treatment with cDDP (3, 5, 6, 7 mg $kg^{-1}$) (■) and MD24 rods (containing 5, 7 and 9 mg $kg^{-1}$ cDDP) (●). Mean ± s.d.

i.p. cDDP bolus injection of 5 mg $kg^{-1}$. These data indicated that cDDP formulated in a slow release system reduced renal toxicity. An explanation might be the lower $C_{max}$ for cDDP in plasma after treatment with MD24. Current evidence in humans suggests that single courses of cDDP in the range of 50 mg $m^{-2}$ body surface area will produce reversible renal toxicity in one quarter to one third of the patients treated (Highly *et al.*, 1973; Madias & Harrington, 1978). A similar incidence of reversible toxicity was demonstrated with the regimen of 20 mg $m^{-2}$ body surface area administered for five days (Daugaard *et al.*, 1987; Daugaard & Abildgaard, 1989). This means that more cDDP can be administered without increasing renal toxicity if Pt peak concentrations in plasma are reduced. The same phenomenon is described in this paper when cDDP was built into rods leading to a prolonged release and lower Pt peak concentrations.

The antitumour response was measured by survival of tumour bearing rats. Two different treatment schemes were compared, i.e. equitoxic doses of free cDDP (3 mg $kg^{-1}$) and cDDP (5 mg $kg^{-1}$) incorporated into MD24 rods, and equimolar doses of free cDDP (5 mg $kg^{-1}$) and cDDP (5 mg $kg^{-1}$) incorporated into MD24 rods. Ten rats were treated with equitoxic doses 3 mg $kg^{-1}$ cDDP or 5 mg $kg^{-1}$ cDDP incorporated into MD24 rods. The peak creatinine levels after 3 mg $kg^{-1}$ cDDP and MD24/5 rods were 121 ± 10 µM and 110 ± 22 µM respectively. A third group of ten rats was treated with 5 mg $kg^{-1}$ cDDP, in which creatinine levels in plasma were 320 ± 23 µM, i.e. almost a 3-fold increase in comparison with the same cDDP dose incorporated into MD24 rods. All treatments increased survival time compared with the conrol group ($P < 0.001$) (Figure 3). Further these data demonstrated no significant differences in survival after MD24/5 treatment compared with an equimolar cDDP (5 mg $kg^{-1}$) treatment. The similar platinum concentration in peritoneal tumours after equimolar treatment (Figure 1) were in line with these survival data. It would be expected, however from a pharmacological point of view, that increasing the cDDP dose from 3 mg $kg^{-1}$, administered as a free cDDP, to 5 mg $kg^{-1}$ incorporated in a slow release system would lead to an enhanced tumour exposure and be consequently followed by a better tumour response. A possible explanation why this did not occur might be the fact that the volume of the installed fluid was cleared too rapidly from the peritoneal cavity. At the time of installation of the MD24 rods, 20 ml of fluid is present, while 15 ml of the 20 ml will be cleared within 6 h. (Los *et al.*, 1989*a*). Six hours after installation, when 55% of the drug had been released from the rod, the volume of the remaining fluid (5 ml) may be too small to guarantee a homogeneous distribution of cDDP in the peritoneal cavity (Rosenshein *et al.*, 1978; Dunnick *et al.*, 1979). This would mean that not all tumours in the peritoneal cavity were exposed to the same quantity of drug, leading to a smaller tumour response than expected based on the higher dose.

In conclusion, renal toxicity, due to cDDP treatment, could be reduced by using a slow drug release system implanted intraperitoneally. The antitumour response, however, did not increase after treatment at an equitoxic level, leaving the therapeutic ratio unchanged. Inhomogeneous peritoneal drug distribution may have compromised the result with the slow release treatment.

We would like to thank Dr O. Dalesio for the statistical analysis, Dr Adrian Begg for helpful criticisms and Nel Bosnie for expert technical assistance. This work was supported by Grants NKI 86-5 and NKI 88-2 from the Dutch Cancer Society.

### References

CAMPBELL, A.B., KALMAN, S.M. & JACOBS, C. (1983). Plasma platinum levels: relation to cisplatin dose and nephrotoxicity. *Cancer Treat. Rep.*, **67**, 169.

DAUGAARD, G. & ABILDGAARD, U. (1989). Cisplatin nephrotoxicity. *Cancer Chem. Pharmacol.*, **25**, 1.

DAUGAARD, G., STRANDGAARD, S., HOLSTEIN-RATHLOU, N.H. & 4 others (1987). The renal handling of sodium and water is not affected by the standard dose cisplatin treatment for testicular cancer. *Scand. J. Clin. Lab. Invest.*, **47**, 455.

DEURLOO, M.J.M., BOHLKEN, S., KOP, W. & 4 others (1990). Intratumoral administration of slow release formulations with cisplatin. I: tumour response and toxicity. *Cancer Chem. Pharmacol.* (in press).

DUNNICK, N., JONE,S R., DOPPMAN, J., SPEYER, J. & MYERS, C.E. (1979). Intraperitoneal contrast infusion for assessment of intraperitoneal fluid dynamics. *Am. J. Radiol.*, **133**, 221.

HIGHLY, D.J., WALLACE, H.J. & HOLLAND, J.F. (1973). Cis-diamminedichloroplatinum(II) (NSC-119875): a phase I study. *Chemother. Rep.*, **57**, 459.

LOKICH, J.J. (1980). Phase I study of cis-diamminedichloroplatinum (II) administered as a constant 5-day infusion. *Cancer Treat. Rep.*, **64**, 905.

LOO, T.L., HALL, S.W. & SALEM, P. (1978). Clinical pharmacological and toxicological studies of cis-diamminedichloroplatinum (II)(DDP) by continuous intravenous infusion. *Biochimie*, **60**, 1067.

LOS, G., MUTSAERS, P.H.A., VAN DER VIJGH, W.J.F., BALDEW, G.S., DE GRAAF, P.W. & MCVIE, J.G. (1989*a*). Direct diffusion of cis-diamminedichloroplatinum(II) in intraperitoneal rat tumors after intraperitoneal chemotherapy: a comparison with systemic chemotherapy. *Cancer Res.*, **49**, 3380.

LOS, G., RUEVEKAMP, M., BOSNIE, N., DE GRAAF, P.W. & MCVIE, J.G. (1989*b*). Intraperitoneal tumor growth and chemotherapy in a rat model. *Eur. J. Cancer Clin. Oncol.*, **25**, 1857.

MADIAS, N.E. & HARRINGTON, J.T. (1978). Platinum nephrotoxicity. *Am. J. Med.*, **85**, 307.

PATTON, T.F., REPTA, A.J., STERNSON, L.A. & BELT, R.J. (1982). Pharmacokinetics of intact cisplatin in plasma. Infusion versus bolus dosing. *Int. J. Pharmacol.*, **10**, 77.

ROSENSHEIN, N., BLAKE, D., MCINTYRE, P. & 4 others (1978). The effect of volume on the distribution of substances installed into the peritoneal cavity. *Gynecol. Oncol.*, **6**, 106.

VERMORKEN, J.B., VAN DER VIJGH, W.J.F., KLEIN, I., GALL, H.E. & PINEDO, H.M. (1989). Pharmacokinetics of free platinum species following rapid, 3-h and 24-h infusions of cis-diamminedichloroplatinum(II) and its therapeutic implications. *Eur. J. Cancer Clin. Oncol.*, **18**, 1069.